# Overview of the perceived risk of transboundary pig diseases in South Africa

**DOI:** 10.4102/jsava.v86i1.1197

**Published:** 2015-05-22

**Authors:** Japhta M. Mokoele, Leana Janse van Rensburg, Shanie van Lochem, Heinz Bodenstein, Jacolette du Plessis, Chris A.P. Carrington, B. Tom Spencer, Folorunso O. Fasina

**Affiliations:** 1 Department of Production Animal Studies, University of Pretoria, South Africa; 2 Limpopo Department of Agriculture, FCM Building Groblersdal, South Africa; 3 National Department of Agriculture, Forestry and Fisheries, South Africa; 4 Preventicare Veterinary Consultancy, Pretoria, South Africa; 5 CS Vet, Menlopark, South Africa

## Abstract

Pig production is one of the most important animal agricultural activities in South Africa, and plays a definite role in providing food security for certain population groups in the country. As with all animal production systems, it is subject to the risk of outbreak of transboundary diseases. In the present overview, evaluations of the perceived risk of selected transboundary animal diseases of pigs, as collated from the willing participants from the provincial veterinary services of South Africa, are presented. A scenario tree revealed that infected but undetected pigs were the greatest perceived threat. The provincial veterinary services, according to participants in the study, face certain difficulties, including the reporting of disease and the flow of disease information amongst farmers. Perceived strengths in surveillance and disease monitoring include the swiftness of sample despatch to the national testing laboratory, as well as the ease of flow of information between the provincial and national agricultural authorities. The four factors were identified that were perceived to most influence animal health-service delivery: transport, access, livestock policy and resources. African swine fever was perceived to be the most important pig disease in South Africa. Because the decentralisation of veterinary services in South Africa was identified as a potential weakness, it is recommended that national and provincial veterinary services need to work together and interdependently to achieve centrally controlled surveillance systems. Regionally-coordinated surveillance activities for certain transboundary diseases were identified as needing priority for the southern African region. It is proposed that an emergency preparedness document be made available and regularly revised according to the potential risks identified on a continuous basis for South Africa.

## Introduction

South Africa has three different sectors of pig farming, namely:

- Commercial, which can be divided into two categories (they are mostly concentrated in the 200 km radius around Pretoria):
  - compartments, which maintain closed herds with high biosecurity and feed commercial pig rations. The pigs are slaughtered at commercial abattoirs
  - other commercial units, which do not buy pigs at auctions, have varying levels of biosecurity, feed commercial pig rations and the pigs are slaughtered at commercial abattoirs.
- Small and semi-commercial units, which have low biosecurity with frequent movements between farms, including auctions, and the rations vary greatly but can include cooked and illegally-fed swill. These farms usually supply local markets and few pigs are slaughtered at abattoirs. These farmers are dependent on the maize price and the farms are mainly situated in the Cape and around the maize belt of South Africa.
- Partially to fully free-range, which are rural and have pigs roaming freely and mostly feeding off scraps that are thrown out by households. The pigs are occasionally confined to protect crops. These pigs are slaughtered informally for special events and contribute to food security for those with a low socio-economic status.

According to the statistics from the South African Pork Producers’ Organisation, the South African commercial pig industry has a current population of 97 532 heads of sows and approximately 7000 boars, the majority of which are resident in the northern provinces of Gauteng, Limpopo, Mpumalanga and the North-west. There are 46 registered pig abattoirs that slaughter approximately 2 million pigs per annum and a total of about 400 registered farmers. The industry also boasts a standing pig population of over 1.63 million and an annual production value of approximately R3 billion ($392 million). It is confirmed that zero risk is impossible, either in an extensive or intensive system of livestock management, and animal intensification is often associated with an increased risk of outbreaks of disease due to an increasing population within a limited land space (Cheneau, El Idrissi & Ward 2004; Fasina *et al*. 2012; Graham *et al*. 2008). South Africa has a very-good-to-excellent border-control system but shares lengthy borders with six contiguous countries; therefore, the possibilities of illegal entry of pigs, pig products, inadvertent importation of genetic material, and movements of pets by immigrants remain, and these constitute another level of risk to the country (Penrith & Thomson 2012).

Recently, at different times, exotic diseases (serotype O foot and mouth disease [FMD], porcine reproductive and respiratory syndrome [PRRS] and classical swine fever [CSF]) have entered South Africa. Outbreaks of FMD, caused by the endemic South African territory (SAT) serotypes, have occurred in cattle in the FMD-free zones, and the impact of these outbreaks to the industry was minimised through the rapid intervention and collaborative efforts of the national and provincial veterinary services (National Department of Agriculture [NDA] 2001). Early in 2012, the African swine fever (ASF) virus left its traditional control area in South Africa to cause outbreaks in the ASF-free areas. Officially, South Africa is free from all of the diseases used in this report.

In the present circumstances of globalisation and trade liberalisation, including the sociopolitical problems in certain countries in Africa, human movement into South Africa will likely continue and, despite strict border control, this can be assumed to be accompanied with the inadvertent importation of animal products (Chaber *et al*. 2010).

Although porcine epidemic diarrhoea (PED) has never been documented in South Africa, its effect in the global pig industry has made the South African Pork Producers Organisation (SAPPO) engage the Department of Agriculture, Forestry and Fisheries in appropriate control for imports, especially concerning live pigs.

Between 1993 and 2009, the South African pig industry, in conjunction with the national veterinary authorities, coordinated 11 rounds of serological and, sometimes, virological testing for pig diseases. Where applicable, duplicate testing was conducted in international reference laboratories to validate the results. Whilst some diseases were routinely included for testing (ASF, FMD, CSF and PRRS), based on expediency and the present emerging threats, others were tested from time to time, based on the needs and recommendations from the industry and the agricultural authorities. Details of these results are available on the SAPPO website (SAPPO 2012).

Although a rapid response enabled the incursions of exotic pig diseases to be controlled, the fact that they may have entered the country after a long period of freedom from those diseases is concerning. The provincial veterinary services are the first line of defence against disease incursions and, therefore, a questionnaire survey was undertaken to obtain an understanding of their capacity to protect the pig industry and to identify any challenges that they might face in this respect.

Therefore, to evaluate the perceived risks of selected transboundary pig diseases in South Africa and to assess the impacts of provincial veterinary services on the national swine herd, in terms of disease surveillance and management system, a scenario tree analysis was used and a questionnaire survey was carried out with voluntary participants (private, provincial and national veterinarians with interests in pig diseases). The results were analysed to arrive at conclusions regarding the perceived risk.

## Material and methods

### Scenario tree analysis and matrix scoring

**FIGURE 1:**
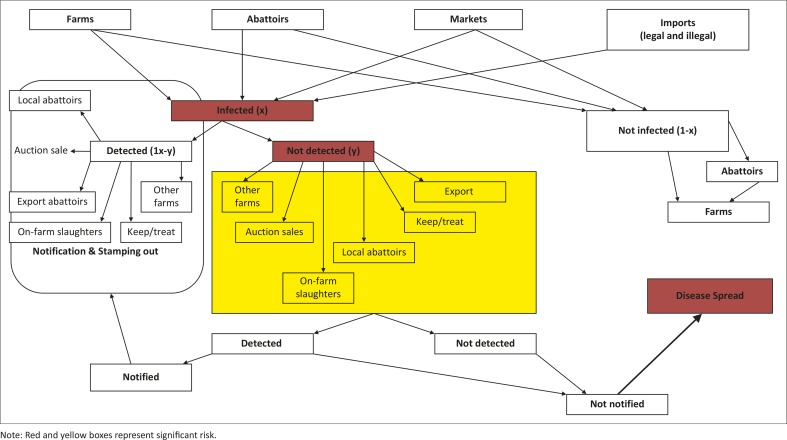
Scenario tree representing the likelihood of disease detection in the transactions and pig movements pathways within South Africa. Note: Red and yellow boxes represent significant risk.

Note: Red and yellow boxes represent significant risk.

As part of the present study, a scenario tree pathway (Figure 1) was developed at a round-table discussion with seven pig veterinarians from South Africa, as well as veterinarians involved in postgraduate training. This scenario tree pathway was based on the veterinary infrastructures at provincial level, the perceived risk of outbreaks, the perceived chance of detection, marketing and abattoir networks, and the import-export system of pigs and their products in South Africa.

**FIGURE 2:**
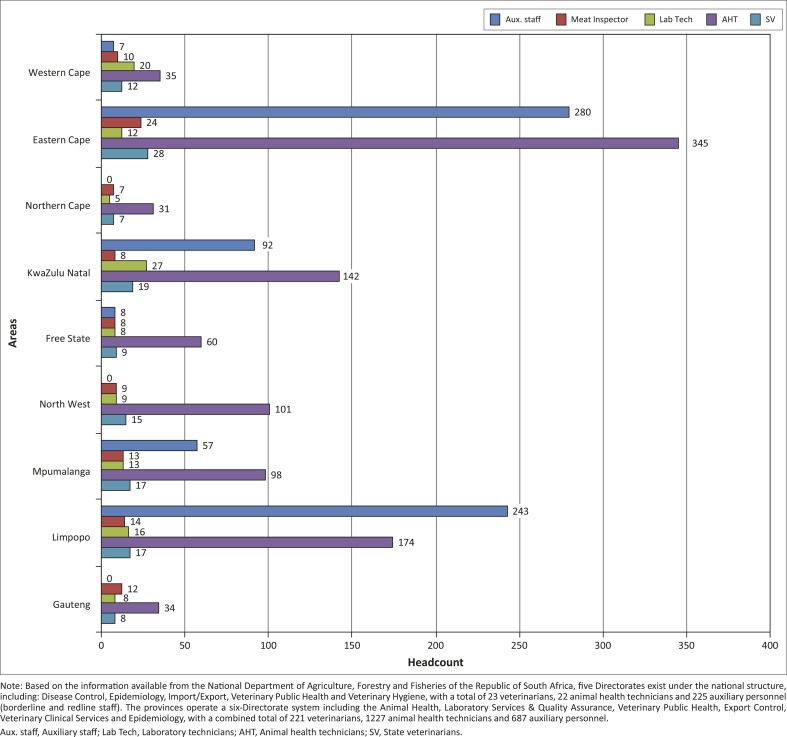
Headcounts of Provincial Animal Health Directorate Veterinary and Paraveterinary professionals and related associates.

Note: Based on the information available from the National Department of Agriculture, Forestry and Fisheries of the Republic of South Africa, five Directorates exist under the national structure, including: Disease Control, Epidemiology, Import/Export, Veterinary Public Health and Veterinary Hygiene, with a total of 23 veterinarians, 22 animal health technicians and 225 auxiliary personnel (borderline and redline staff). The provinces operate a six-Directorate system including the Animal Health, Laboratory Services & Quality Assurance, Veterinary Public Health, Export Control, Veterinary Clinical Services and Epidemiology, with a combined total of 221 veterinarians, 1227 animal health technicians and 687 auxiliary personnel.

In addition, a colour-coded risk-estimate table, which was based on a table devised for estimating the risks of GMOs (Office of Gene Technology Regulator [OGTR] 2005), was provided for the pig veterinarians to comment on their perception of the likelihood of occurrence and level of risk posed by 11 selected pig diseases (ASF, FMD, Porcine circovirus type 2 (PCV), CSF, PRRS, brucellosis, swine influenza (SI), porcine respiratory coronavirus (PCRV), transmissible gastroenteritis (TGE), swine vesicular disease (SVD) and Aujeszky’s disease (Figure 2). Prioritisation criteria for the diseases were set according to the previously established guidelines on epidemiology, prevention/control, effect on economy/trade, disease characteristics/zoonotic potential, and the effect on the society (Humblet *et al*. 2012).

Aux. staff, Auxiliary staff; Lab Tech, Laboratory technicians; AHT, Animal health technicians; SV, State veterinarians.

A set of questions, which were adapted from the work of Geering and Lubroth (2002), guided the questioning and included the following:

- What is the current geographic distribution and incidence of the disease nationally, regionally and internationally?
- How close are the susceptible species to the areas of significant disease threat?
- Is the disease spreading to naive countries, or does it have a static status?
- Is there a vaccine available or any protective strategy?
- Is the disease of significance in the neighbouring countries and what are the capacities of their veterinary services?
- Are there national parks, or wild and feral animal populations that can serve as carriers or reservoir hosts?
- How is movement controlled and how are pig marketing structures within the country monitored?
- Are there significant import risks?
- Are affected animals likely to find means of crossing quarantined areas and barriers set by the veterinary authorities?

The perceived economic impacts and the long-term consequences of the diseases were considered in the final weighting and categorisation of each disease. Based on the placement of each disease on the risk-estimate table by individual pig veterinarians, the scores for the 11 identified pig diseases were aggregated and mean values were obtained for each. A disease radar chart (spider web) was produced using individual weighting for the diseases.

### Questionnaire survey

A survey was conducted among members of the provincial veterinary services between 20 July and 10 August 2012, it was based on a questionnaire that was developed and tested amongst six pig veterinarians to assess the ease of understanding of the question and validity of the testing instrument. Based on the responses, the self-rated questionnaire was adjusted and sent by email; this was supported in certain instances by physical circulation of paper copies during the Southern African Society for Veterinary Epidemiology and Preventive Medicine (SASVEPM) congress meeting in August 2012. The aim of the questionnaire was to collate the perception on the ease of administration of pig-related veterinary services at provincial level, based on each participant’s opinion, and to critically evaluate such services provided in the provinces.

The inclusion criteria for participants were as follows: (1) being a state veterinarian or a pig consultant, and (2) having had provision of pig-related veterinary services within the last 3 years. The exclusion criteria were: (1) being part of the study-group membership, (2) specialisation in other species or (3) having other interests that could potentially bias the study. The six veterinarians who were used to test the questionnaire instrument were similarly excluded from participating in the final questionnaire. All participants willingly consented to be part of the study. A total of 65 questionnaires were administered and 57 (87.69%) were returned; incomplete responses or multiple responses rendered another 12 to be unusable. A total of 45 responses (69.23%) were entered into an excel data sheet (Microsoft, USA) and a descriptive analysis and a rotated factor loading analysis (RFLA) were conducted on the data using the online WESSA statistical software (Wessa 2012). Weighting values of ≥ 0.500 were taken as significantly positively or negatively correlated in the RFLA.

## Results

### Scenario tree analysis

Infected but undetected pigs were perceived to represent the greatest animal-associated risk to the South African pig industry. The general opinion was that the perceived risk of transboundary and infectious pig diseases may originate from the farms, markets and abattoirs, or from import sources. In each case, the disease can either be detected and eliminated or pass unnoticed, in which case a further extension of the risk is probable. The undetected cases may then be reintroduced to other farms, sold at auctions, spread locally through home slaughter or be slaughtered at the local abattoir. An infectious animal or disease may possibly be sent to an export abattoir (an unlikely event due to the stringent conditions that need to be satisfied to meet the requirements of an export abattoir, as well as the low volume of pork destined for export). Detection at this level may stop further spread of the disease; however, in the case of non-detection, the industry may suffer widespread outbreaks following this event (see Figure 1).

### Veterinary services

The low incidence of the reporting of unusual deaths and the flow of livestock-disease information between farmers and veterinary services were the most important perceived limitations to the effective rendering of pig-related veterinary services in South Africa. The rapidity of sample transportation to the national testing laboratory and the flow of information between the provincial and national veterinary authorities were perceived to be the least difficult operations to perform (Table 1a). Of the participating veterinarians, 31.00% agreed that, in their opinion, an effective pig disease-prevention system was in place within the provinces, whilst 35.71% stated that veterinarians were always present at pig auction sites and markets (Table 1b). A total of 76.19% perceived that the routine inspection of pork and other pig products was performed at all abattoirs, but only 21.00% confirmed that enough funds were made available annually for the effective provision of all veterinary services. Logistic problems and inadequate manpower were perceived to be significant deficiencies that needed immediate attention within the provinces (74.42% and 79.07%, respectively). Just over 33.00% perceived that all the basic equipment needed for effective pig-related veterinary services was available and 21.43% felt that they could conduct penside/rapid diagnostic tests due to a perceived availability or lack of the available resources. Veterinary drugs were perceived to be easily accessible at provincial level (51.16%) but black-market drugs and vaccines were perceived to be somewhat of a problem (34.15%). Supporting infrastructures were perceived to be available within the provinces (47.50%); however, movement control of pigs within certain provinces was perceived to be deficient. There was also an opinion that more government attention should be paid to veterinary services at provincial level, because only 21.43% of the responses perceived that it was currently adequate. Overall, the availability of the different role players in the animal health services differ from province to province (Figure 2).

**TABLE 1a:** Self-rated questionnaire on the ease of providing pig-related veterinary services in South African provinces.

Provincial capacities	Scoring on a Likert scale					Mean score obtained	Difficulty rank based on mean
		Very easy	Easy	Moderate	Difficult			Very difficult		
**Capacity of the provinces to:**						
Collect reports of unusual death patterns from farmers	1	2	3	4	5	3.77 ± 0.91	1
Livestock disease information flow between pig farmers	1	2	3	4	5	3.68 ± 1.04	2
Do trace back and trace forward	1	2	3	4	5	3.26 ± 1.11	3
Initiate internal movement control	1	2	3	4	5	2.95 ± 1.31	4
Collaborate with other provinces on disease diagnosis and surveillance	1	2	3	4	5	2.66 ± 1.13	5
Send reports and contact all contiguous provinces and provinces along the routes of animal movements	1	2	3	4	5	2.65 ± 1.19	6
Perform passive surveillance	1	2	3	4	5	2.60 ± 1.16	7
Perform active surveillance	1	2	3	4	5	2.51 ± 1.12	8
Gain rapid access to all areas/jurisdiction under veterinary control	1	2	3	4	5	2.33 ± 1.06	9
Rapidly collect samples in outbreak situations	1	2	3	4	5	2.27 ± 1.04	10
Rapidly send samples to the national veterinary testing laboratory (OVI) for disease confirmation	1	2	3	4	5	2.14 ± 1.05	11
Contact and inform the National Veterinary Service of outbreaks	1	2	3	4	5	1.95 ± 0.96	12

Note: Rank 12 is the least difficult activity, whilst Rank 1 is the most difficult activity, based on the state veterinarians’ perception ranking. Perception ranking: 1 to 2.5 is very easy to moderate; 2.51 to 3.5 is moderate to difficult; and 3.51 to 5.0 is difficult to very difficult.

Significant difference exists between the 10 most difficult pig-related veterinary services provided by the veterinary officers (*p*-value < 0.0001; *F*statistics = 9.916).

**TABLE 1b:** Evaluation of pig-related veterinary services within the South African provinces.

Services	Total	Yes	%	Range at 95% CI	*N*	%	Range at 95% CI
Livestock (pig) disease prevention system is in place	42	13	30.95	18.42, 46.03	29	69.05	53.97, 81.58
Province Veterinary Department have veterinary officers/ animal health assistants in all the pig auction sites and markets	42	15	35.71	22.39, 50.95	27	64.29	49.05, 77.61
Province Veterinary Department do routine inspections at the abattoir	42	32	76.19	61.65, 87.23	10	23.81	12.77, 38.35
Province has enough budgets for all veterinary services per annum	43	9	20.93	10.72, 34.95	34	79.07	65.05, 89.28
Province encounters logistic problem associated with veterinary services within the province	43	32	74.42	59.89, 85.75	11	25.58	14.25, 40.11
Province has a shortage of manpower for veterinary services	43	34	79.07	65.05, 89.28	9	20.93	10.72, 34.95
Basic veterinary equipment available in the province	42	14	33.33	20.39, 48.51	28	66.67	51.49, 79.61
Conduct pen-side tests for animal diseases within the province	42	9	21.43	10.99, 35.69	33	78.57	64.31, 89.01
Veterinary drugs easily accessible within the province	43	22	51.16	36.39, 65.78	21	48.84	34.22, 63.61
Black-maketeering is a problem for veterinary drugs within the province	41	17	34.15	20.93, 49.54	27	65.85	50.46, 79.07
Supporting infrastructures within the province for veterinary services is available	40	19	47.50	32.47, 62.88	21	52.50	37.12, 67.53
Livestock (pigs) move freely within the province	43	7	16.28	7.41, 29.57	36	83.72	70.43, 92.59
Level of government attention to veterinary issues adequate within the province	42	9	21.43	10.99, 35.69	33	78.57	64.31, 89.01

Note: It should be noted that differential capacities exist between the provinces and certain provinces have significant strength in animal disease surveillance compared with others. The outcomes of the survey represent the overall mix of all opinions amongst the provinces, based on the subjects’ willingness to participate. Regularity of abattoir visits varies amongst provinces. Certain provinces have organised the veterinary services in a way to separate the Veterinary Public Health Section/Unit, who pay more regular visits to the abattoir within such provinces. Important endemic diseases in certain locations include: ASF, erysipelosis, sarcoptic mange, leptospirosis, bacteria septicaemia, production and management diseases and *Taenia solium*, in that order. It should be noted that ASF is not endemic in all parts of South Africa, but is restricted to the control zones within KwaZulu Natal, Mpumalanga, Limpopo and the North West.

Mid *p*-exact percentages and ranges were calculated at 95% confidence interval (95% CI).

### Latent variables for risk of pig diseases

Factors that were identified as influencing the risk of pig diseases in South Africa included: transport, access, livestock policy/*Animal Diseases Act* (Act 35 of 1984) implementation and resources/budget allocations (Table 2).

**TABLE 2:** Factor analysis of the latent variables and their corresponding indicators using Rotated Factor Loadings (Varimax).

Variables	Factor 1	Factor 2	Factor 3	Factor 4
Province collects reports of unusual death patterns from farmers	0.088	0.754	–0.048	–0.093
Livestock disease information flows between pig farmers in the province	0.118	0.667	0.302	–0.136
Province performs trace back and trace forward	0.361	0.585	–0.020	–0.100
Province initiates internal movement control	0.748	–0.006	0.182	–0.389
Province collaborates with other provinces on disease diagnosis and surveillance	0.386	0.368	0.076	–0.393
Province sends reports and contacts all contiguous provinces, and provinces along the routes of animal movements	0.702	0.162	–0.025	0.066
Province performs passive surveillance	0.707	0.220	–0.008	–0.0560
Province performs active surveillance	0.595	0.273	–0.251	0.454
Province gains rapid access to all areas/jurisdiction under veterinary control	0.250	0.775	–0.254	–0.024
Province rapidly collects samples in outbreak situations	0.395	0.577	–0.346	0.035
Province rapidly sends samples to the national veterinary testing laboratory (OVI) for disease confirmation	0.755	0.119	–0.274	0.073
Province contacts and informs the National Veterinary Service of outbreaks	0.601	–0.130	–0.413	–0.226
Livestock (pigs) disease prevention system is in place	–0.088	–0.021	0.725	0.046
Provincial Veterinary Department has vet officers/animal health assistants in all the pig auction sites and markets	–0.129	–0.048	0.528	0.214
Provincial Veterinary Department performs routine inspections at the abattoirs	0.089	–0.432	0.362	–0.125
Provincial Veterinary Department has adequate budgets for vet services/annum	–0.139	–0.186	0.393	0.542
Provincial Veterinary Department encounters logistic problem associated with veterinary services within the province	0.162	0.202	0.125	–0.655
Provincial Veterinary Department has a shortage of manpower for vet services	0.125	0.326	0.271	–0.031
Basic veterinary equipment is available within the province	0.087	–0.121	0.428	0.601
Pen-side tests for animal diseases are conducted within the province	0.017	–0.201	0.571	–0.018
Veterinary drugs are easily accessible within the province	0.068	–0.015	0.107	0.784
Black-maketeering of veterinary drugs are major issues within the province	0.321	0.083	0.204	–0.036
Supporting infrastructures for veterinary services within the province are available	0.046	–0.530	0.090	0.189
Livestock move unhindered within the province	0.152	0.126	0.209	–0.030
There is an adequate level of government attention paid to veterinary issues within the province	–0.356	–0.029	0.585	0.394

Note: Extraction method, factor analysis; Rotation method: Varimax with Kaiser normalisation. The loadings of indicators building the factors (larger than 0.5) are in bold fonts.

Factor 1, transport-related factor; Factor 2, access-related factor; Factor 3, livestock policy/*Animal Diseases Act*-related factor; Factor 4, resource/budget-related factor.

The first factor was transport related, which is primarily a measure of the rapidity of sending samples to the national laboratory. However, this factor is also a measure of internal movement control, active and passive surveillance and effectiveness of contacting the contiguous provinces and the national veterinary services in an outbreak situation. As one variable increases, the other variables tend to increase correspondingly; thus, a failure in any one of the factors above will tend to lead to a failure of the correlated factors (Table 2).

The second factor is access related, which is primarily a measure of gaining rapid access to all areas under veterinary jurisdiction, but also of collecting reports of unusual deaths from farmers, flow of livestock information between farmers, performing trace-back and trace-forward, and collecting samples rapidly in an outbreak situation. This variable is negatively correlated with the availability of supporting infrastructures for veterinary services within the province (the more these infrastructures are available, the less they will be the dependence on the other factors that support access). Failure of any of the above services, however, will tend to have a negative effect on the effectiveness of the other correlated factors and an inverse effect on supporting infrastructures (Table 2).

The livestock policy/*Animal Disease Act* implementation-related factor is the third important variable that influences the risk of pig diseases, and it is primarily a measure of the perceived availability of pig-disease-prevention measures in place, the perception of whether government is paying adequate attention to veterinary services, the perceived availability of pen-side and rapid tests at provincial level, and the perceived attendance of provincial veterinary officers at all pig auctions and markets. Failure of these services will produce a negative effect on policy supporting livestock services and drive pig disease intensification in South Africa (Table 2).

The fourth factor (resources/budget-related variable) is primarily a measure of the perceived accessibility of veterinary drugs within the province, availability of basic veterinary equipment and adequate annual budget allocation for the provincial veterinary offices.

### Matrix scoring for risk of specific diseases

**FIGURE 3:**
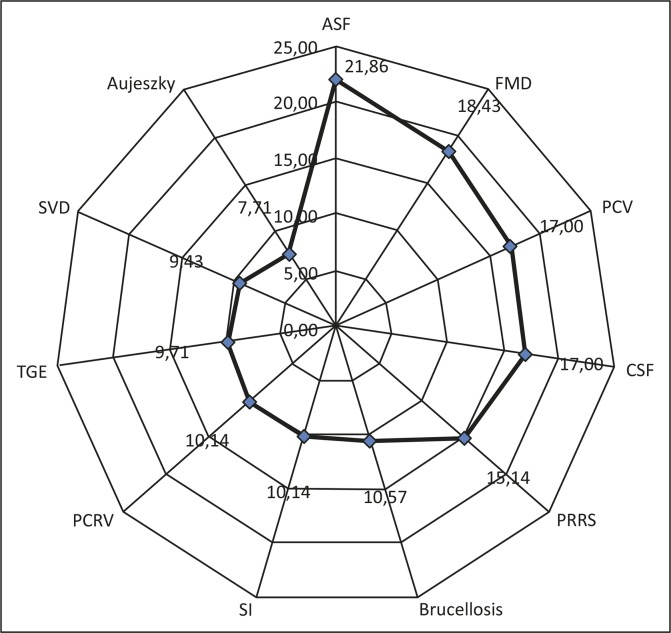
Radar chart (spider-web) of important pig diseases based on experts’ ratings.

Based on the expert ratings, ASF remains the most significant pig disease, in terms of perceived risk of outbreak and economic impacts in South Africa (21.86/25), followed by FMD (18.43/25). Porcine circovirus 2 (PCV 2) and CSF were both placed third, with a score of 17.00/25 each, and Aujeszky’s disease was the least significant disease, with a score of 7.71/25 (Figure 3). Other diseases of importance for South Africa, based on perceived risk of introduction, include: PRRS, *Brucella suis*, swine influenza, transmissible gastroenteritis (TGE) and porcine respiratory coronavirus (Figure 3).

## Discussion

In the present analysis, the perceived risks and likely routes of entry of transboundary and infectious pig diseases into the South African pig population have been identified. These include the following: internal pig movement routes (especially in some restricted locations) and associated informal markets/pig auctions, restricted home slaughter of pigs and inadequate surveillance for infectious diseases amongst smallholder farms.

The lack of complete reporting within the country and also in the neighbouring countries will continue to pose a risk of disease introduction and spread to South Africa, with a potentially significant impact on livestock and animal biodiversity, as well as human livelihoods (Chaber *et al*. 2010; Penrith & Thomson 2012). In view of the routine testing systems and rigid import requirements, the risks associated with legal importation of animal products are minimal but should not be ignored.

The perceived lack of specific pig-disease-prevention systems, logistic problems, inadequate budget allocation, manpower shortages, lack of infrastructure, poor government attention to veterinary matters, and other associated factors have been identified as areas of perceived weakness in the current provincial veterinary services system (Table 2). It would appear that the strengthening of some of these services will have a positive effect on corresponding and related services, and will generally improve service delivery in animal health. Bayissa and Bereda (2009) previously identified these challenges as major limitations to the effective cross-border animal-health management systems at the Ethiopia/Kenya borders. In addition, Benet, Dufour and Bellemain (2006) had previously stated that logistics, poor financial resources, insufficient involvement of livestock producers and private veterinarians are major limitations to effective veterinary services worldwide.

Whilst it is generally agreed that the the South African national and provincial veterinary services have significant strength in certain areas of operations, especially expertise and facilities, compared with many other southern African countries, the delivery of livestock services in South Africa currently runs in parallel with them. The national veterinary authority has a mandate to coordinate disease control and prevention activities nationally, and the provincial authorities coordinate and implement similar activities at their levels. This development allows for the weakening of the whole system of animal disease control, especially in situations where the same degree of coordination is not ensured by all provinces or when operational funds are not immediately available in certain provinces. Control of some rapidly spreading diseases may also benefit from a single line of command for effective surveillance and monitoring. This would need to have a more centrally controlled surveillance system/line of command, especially with regard to rapidly spreading infectious diseases. Veterinary services should strengthen interdependence amongst the provinces and the national services, including an enabling law that mandates a province with infection to inform other contiguous provinces.

There is a need for the reorganisation of auctions, in terms of improvement of the biosecurity systems and provision of holding facilities, as well as education of farmers on the dangers of returning unsold animals back to the farm of origin. It may also be necessary to build an open abattoir close to such auction facilities to encourage the sale-for-slaughter of animals that are not bought for the purpose of introduction to other farms. Similarly, veterinary control posts and possibly mini-laboratories that can provide rapid and efficient services are needed around such auction facilities to ensure that infectious animals are not passed into the system, with consequent spread of diseases.

It is important to fill the vacant positions of veterinary practitioners and encourage the system to retain trained veterinarians, open the system to foreign-qualified veterinary professionals and restructure the state veterinary services to have a more effective service delivery system.

African swine fever and FMD remain significant threats to the South African pig population, in view of their geographical distribution amongst the wild populations (warthogs and buffaloes) in the Kruger National Park. Although the distribution of these diseases is fairly static in these regions, changes in environmental ecology and the movement of vectors, as well as human and vehicular movements, may be expanding the reach of these infectious agents. It will be imperative to check the geographical boundaries (red lines) set for these diseases through routine border-line surveillance and intensify it where disease exists. Disease information from border countries where certain animal diseases are endemic must also be routinely monitored, so that the current status is known and a comprehensive animal healthcare plan can be made for South Africa. In addition, efforts must be made to ensure that the diseases are kept within the endemic areas, because the eradication of ASF and FMD is impossible due to the wildlife reservoirs of the infection. Joint surveillance and monitoring of these diseases in the Southern African Development Community (SADC) countries will therefore be critical (Otte, Nugent & McLeod 2004; Penrith & Thomson 2012).

Although PCV-2 may be an economically important disease in South Africa, to date, no specific surveillance has been conducted to validate the current status because the disease is thought to be ubiquitous and present in most countries. Vaccination against the disease is carried out in several of the commercial and semi-commercial farms to prevent its effect on productivity. Classical swine fever and PRRS are routinely surveyed by the industry and, to date, apart from the historical outbreaks in the Western and Eastern Cape region, the country has largely been free. It will be important to continue to monitor the situation of these diseases as events unfold, although officially they have been eradicated since 2009. It is noteworthy that the numbers and distribution of veterinarians and paraveterinarians, who were available for frontline surveillance and emergency services in the regions where these diseases were a problem in the past, are strategic and significant (Figure 3).

Risk profiling of pig diseases and those of other animals, as well as prioritising the diseases, will need to be periodically conducted and evaluated. The reorganisation of laboratory services, in terms of strength and capabilities for better service delivery, may be imperative to meet the need for urgency of sample handling and provision of frontline services prior to more detailed confirmatory diagnoses at the central laboratories. There are needs for location-specific and targeted training of provincial and national veterinary officers and their staff, as well as production-related and biosecurity-type training for farmers.

Although enabling policies and some funding currently exist for veterinary services at national and provincial level, there will be a need to further strengthen these areas and put in place the necessary institutional reforms to improve these issues. The private veterinary services must be encouraged to be involved in clinical veterinary healthcare delivery systems, especially at the smallholder and rural levels, and subcontracting of services using public-private partnership platforms that will be effectively managed at such levels should be promoted by the authorities (Cheneau *et al*. 2004; Holden, Ashley & Bazeley 1996).

It is also important to consider a system where multi-pronged operations can run in parallel towards a single improved pig health system, as was previously analysed in England by Stärk and Nevel (2009). Such analyses must place emphasis on areas of strengths and weaknesses of the industry-led, government-led and public-health-led surveillance activities, and to consider areas of bridging, joint operations and differences.

## Disease control in South Africa and the implications for other Southern African territories

The key to success in any transboundary animal disease management is early detection when an outbreak occurs and a good network of animal disease information. It is imperative to regularly evaluate and improve the existing nationally coordinated control system that can jump start into action through either the passive or active disease surveillance systems, as well as have a trigger system established at the farm, abattoir or animal route-level.

Since South Africa shares inclusive borders with some countries (Lesotho and Swaziland) and extensive borders with certain other southern African countries, and a high degree of human and vehicular movements are exchanged daily along these borders, it is critical for the national and provincial veterinary authorities in South Africa to have regular updates and monitor reports of diseases in these other countries so that they can continually improve veterinary services and respond timeously to potential animal health threats.

It is well established that the presence and spread of transboundary diseases of animals in a country is a constant threat to pig and other animal populations in contiguous countries, and this situation has been well exemplified by the ASF epidemic that has spread through the West African subregion since 1996 (El-Hicheri 1998; Penrith *et al*. 2009); therefore, a regionally coordinated control of certain diseases that does not respect geographical boundaries is necessary. Penrith and Thomson, (2012) recently emphasised such a need, based on a study in the Kavango-Zambezi Transfrontier Conservation Areas (TFCA), which comprises five southern African countries. Such regionally coordinated disease-control efforts should evaluate the animal disease capabilities of each partner country, consider the assessment of disease threats country by country, have a standby emergency response team and create a shared database of disease that is easily accessible and utilised by all partners (Frawley 2003).

## Need for emergency preparedness documents

Because it is critically important to break the transmission cycle of any rapidly spreading disease agent as soon as it is recognised in a country, in the case of a suspicion of any transboundary disease there is a need to enter into agreements with established farms and abattoirs to perform immediate notification and trigger the alert system. Such agreements should be revised periodically. Consideration should be given to emerging and small scale farmers, in terms of the provision of animal health services, input supplies, marketing and other veterinary services, because these individuals will continue to utilise livestock resources, including pigs, as a form of investment with good returns and this may ultimately impact on animal health.

It is important to fully integrate those emerging, small-scale and middle-scale farmers into the industry, give them adequate livestock education, encourage the importance of farm-level biosecurity and encourage disease reporting among themselves. The option of compulsory exclusion of this group from the South African pig industry will only prevent adequate reporting from smallholder farmers and negatively affect the pig industry.

Finally, a contingency document will need to be put in place at national level and adapted at provincial level to strengthen disease reporting by farmers, active disease surveillance and response strategies. Such a document must be subjected to timely reviews and be tested by simulations that must be carried out from time to time.

## Conclusion

Risk and threat to animal health will exist as long as animal production continues, and limiting the effect will be dependent on a combination of factors, some of which have been identified in the present report. Continual improvement in the animal health strategies that exist within the Republic is advocated, as is the inclusion of smallholder farmers in comprehensive biosecurity programmes. Although budget restrictions, growing fiscal deficits and insufficient funds will continue to militate against effective animal health services in African countries (De Haan & Nissen 1985), the careful balancing and effective utilisation of scarce resources for efficacious delivery of animal health services will need to be paramount. There is a need for a regionally coordinated control of certain diseases because the geographical boundaries appear to be blurred (Penrith & Thomson 2012).

## References

[CIT0001] Bayissa B. & Bereda A., 2009, ‘Assessment of veterinary service delivery, livestock disease reporting, surveillance systems and prevention and control measures across Ethiopian/Kenya border’, A report submitted under the Enhanced Livelihoods in Southern Ethiopia (ELSE) project, viewed 06 October 2012, from http://www.disasterriskreduction.net/fileadmin/user_upload/drought/docs/Assessment_of_livestock_health_across_Ethio-kenya_border.pdf

[CIT0002] Benet J.J., Dufour B. & Bellemain V., 2006, ‘The organisation and functioning of veterinary services: Result of a 2005 survey of member countries of the World Organisation for Animal Health’, *Revue scientifique et technique, Office International des Épizooties* 25(2), 739–761. 17094708

[CIT0003] Chaber A.-L., Allebone-Webb S., Lignereux Y., Cunningham A.A. & Rowcliffe J.M., 2010, ‘The scale of illegal meat importation from Africa to Europe via Paris’, *Conservation Letters* 3(5), 317–321. 10.1111/j.1755-263X.2010.00121.x

[CIT0004] Cheneau Y., El Idrissi A.H. & Ward D., 2004, ‘An assessment of the strengths and weaknesses of current veterinary systems in the developing world’, *Revue scientifique et technique, Office International des Epizooties* 23(1), 351–359. 10.20506/rst.23.1.1489 15200109

[CIT0005] De Haan C. & Nissen N.J., 1985, ‘Animal health services in Sub-Saharan Africa: alternative approaches’, World Bank Technical Paper no. 44 The World Bank, Washington DC.

[CIT0006] El-Hicheri K., 1998, ‘Emergency Assistance on control and eradication of an outbreak of African swine fever in Western Nigeria’, Report of the FAO Consultancy Mission to Nigeria, TCP/NIR/7822(E), FAO, Rome, December, 1998.

[CIT0007] Fasina F.O., Ali A.M., Yilma J.M., Thieme O. & Ankers P., 2012, ‘The cost-benefit of biosecurity measures on infectious diseases in the Egyptian household poultry’, *Preventive Veterinary Medicine* 103, 178–191. 10.1016/j.prevetmed.2011.09.016 21982688

[CIT0008] Frawley P.T., 2003, ‘Review of rural veterinary services’, *A report submitted to the Commonwealth of Australia*, viewed 07 October 2012, from http://www.ava.com.au/sites/default/files/documents/Other/Frawley%20report.pdf

[CIT0009] Geering W.A. & Lubroth J., 2002, Preparation of Foot-and-mouth Disease Contingency Plans, FAO Animal Health Manual No. 16 Food and Agriculture Organization of the United Nations, Rome, Italy, viewed 15 December 2012, from http://www.fao.org/docrep/006/Y4382E/y4382e00.htm

[CIT0010] Graham J.P., Leibler J.H., Price L.C., Otte J.M., Pfeiffer D.U., Tiensin T. et al., 2008, ‘The animal–human interface and infectious disease in industrial food animal production: Rethinking biosecurity and biocontainment’, *Public Health Report* 123, 282–299. 10.1177/003335490812300309 PMC2289982 19006971

[CIT0011] Holden S., Ashley S. & Bazeley P., 1996, ‘Livestock in development: Improving the delivery of animal health services in developing countries’, A report to the Overseas Development Administration of the United Kingdom, viewed 10 October 2012, from http://theidlgroup.com/documents/ImprovingthedeliveryofAnimalHelathServicesinDevelopingCountries.pdf

[CIT0012] Humblet M.-F., Vandeputte S., Albert A., Gosset C., Kirschvink N., Haubruge E. et al., 2012, ‘Multidisciplinary and evidence-based method for prioritizing diseases of food-producing animals and zoonoses’, *Emerging Infectious Diseases* [serial on the Internet] Apr [*date cited*], viewed 13 November 2012 10.3201/eid1804.111151 PMC3309682 22469519

[CIT0013] National Department of Agriculture (NDA), 2001, ‘Submission to the Foot and Mouth Disease and Other Epizootics Commission of the OIE for the re-instatement of a foot and mouth disease free zone without vaccination’, August 2001, viewed 10 June 2014, from http://www.nda.agric.za/docs/GenPub/fmdreport.htm

[CIT0014] Office of Gene Technology Regulator (OGTR), 2005, ‘*Risk Analysis Framework*’, Department of Health and Ageing, Australian Government, viewed 31 July 2012, from http://www.ogtr.gov.au/internet/ogtr/publishing.nsf/content/raf-3/$FILE/raffinal2.2.pdf

[CIT0015] Otte M.J., Nugent R. & McLeod A., 2004, ‘Transboundary animal diseases: Assessment of socio-economic impacts and institutional responses’, Livestock Policy Discussion Paper no. 9 Food and Agriculture Organization of the United Nations, viewed 15 December 2012, from http://www.fao.org/ag/againfo/resources/en/publications/sector_discuss/PP_Nr9_Final.pdf

[CIT0016] Penrith M.-L., Guberti V., Depner K. & Lubroth J., 2009, Preparation of African swine fever contingency plans, FAO Animal Production and Health Manual (FAO), no. 8 / FAO, Rome (Italy), Animal Production and Health Div., 2009, 80 p.

[CIT0017] Penrith M.-L. & Thomson G., 2012, ‘Analysis of the Status of Transboundary Animal Diseases and Their Control in the SADC Region During the Period 2005-2011, Focusing on the Five Countries that Contribute Land to the Kavango Zambezi (KAZA) Transfrontier Conservation Area (TFCA)’, Technical Report to the Wildlife Conservation Society’s AHEAD Program, viewed 25 July 2012, from http://www.wcs-head.org/workinggrps_kaza.html

[CIT0018] South African Pork Producers’ Organisation (SAPPO), 2012, ‘Serological Tests’, viewed 25 July 2012, from http://www.sapork.biz/functions/animal-health/serological-tests/

[CIT0019] Stärk K.D.C. & Nevel A., 2009, ‘Strengths, weaknesses, opportunities and threats of the pig health monitoring systems used in England’, *Veterinary Record* 165, 461–465. 10.1136/vr.165.16.461 19850852

[CIT0020] Wessa P., 2012, *Free Statistics Software*, Office for Research Development and Education, version 1.1.23-r7, viewed 10 November 2012, from http://www.wessa.net/

